# Primary Pancreatic Mature T-Cell Lymphoma as a Cause of Obstructive Jaundice: A Case Report and Review of the Literature

**DOI:** 10.7759/cureus.40272

**Published:** 2023-06-11

**Authors:** Rodolfo Garza-Morales, Arup Ganguly, Daniela Hernandez, Brandon Cantazaro

**Affiliations:** 1 Internal Medicine, The University of Texas Rio Grande Valley - Doctors Hospital at Renaissance, Edinburg, USA

**Keywords:** r-chop, non-hodgkin lymphoma, obstructive jaundice, pancreatic malignancy, t-cell lymphoma

## Abstract

Primary pancreatic lymphoma (PPL) is an extremely rare type of non-Hodgkin's lymphoma (NHL). It accounts for 0.1% of all lymphomas and less than 1% of pancreatic tumors. Within this subtype, T-cell lymphomas only account for up to 6.7% of pancreatic lymphomas. In this study, we present the case of a 78-year-old Hispanic man who presented with obstructive jaundice associated with a mass within the head of the pancreas; pathologic analysis of the tumor revealed a mature T-cell lymphoma, not otherwise specified (NOS).

## Introduction

Primary pancreatic lymphoma (PPL) is an extremely rare disease. PPL accounts for fewer than 2% of extranodal malignant lymphomas and <0.5% of cases of pancreatic masses [1]. In more than 80% of cases, PPL presents with right upper quadrant abdominal pain; other clinical findings such as B symptoms (e.g., fever, chills, and night sweats) are rare [2-4]. To date, fewer than 150 cases of PPL have been reported in the literature in English [4]. In this report, we present a case of a 78-year-old Hispanic man with primary pancreatic mature T-cell lymphoma as a cause of obstructive jaundice.

## Case presentation

**Figure 1:**
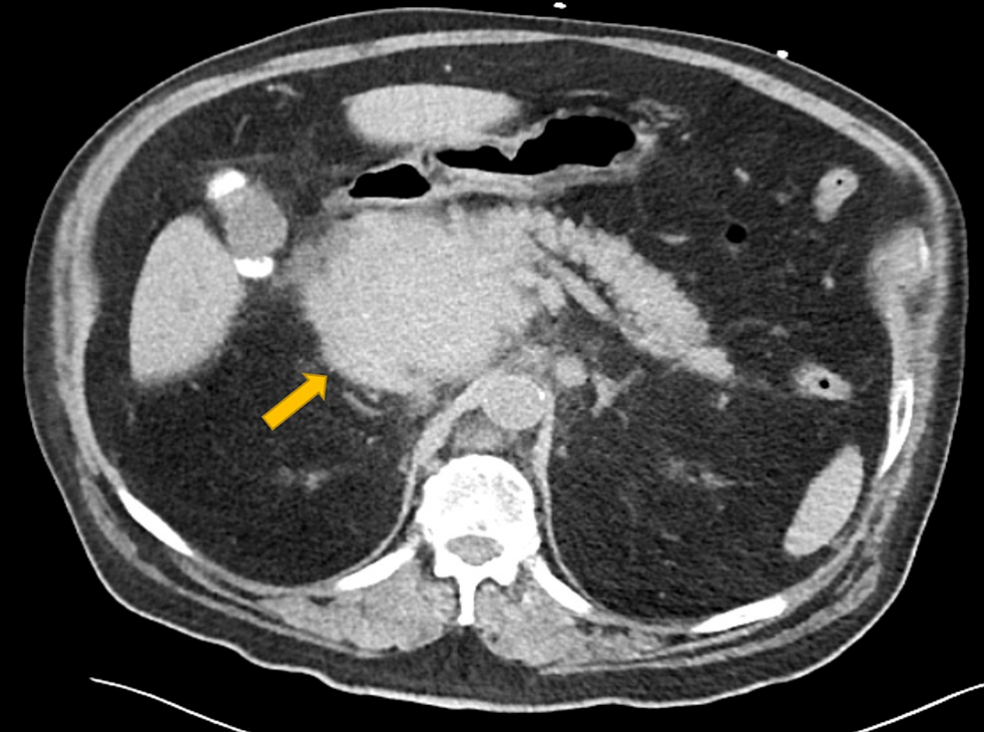
CT abdomen and pelvis without contrast demonstrating large round mass (8.5 x 7.8 cm) within the head of the pancreas (orange arrow) CT: computed tomography

A 78-year-old Hispanic man with a past medical history of hypertension, type 2 diabetes mellitus, and hypothyroidism presented with complaints of three weeks of pain localized to the epigastrium and right upper quadrant, fatigue, jaundice, anorexia, and pruritus. He also endorsed a 20-pound weight loss during the same time period. The laboratory tests on admission revealed total bilirubin of 32.8 mg/dL, alkaline phosphatase (ALP) of 525 IU/L, aspartate aminotransferase (AST) of 124 IU/L, alanine aminotransferase (ALT) of 105 IU/L, as well as lipase of 118 IU/L, carcinoembryonic antigen of (CEA) 3.5 ng/ml, and low CA 19-9 (0.8 IU/ml). Abdominal CT revealed a large mass located in the head of the pancreas measuring 8.5 x 7.8 cm and resulting in extrahepatic/intrahepatic biliary tree dilatation and obstruction, with periaortic and peripancreatic lymphadenopathy (Figure 1).

The patient underwent endoscopic ultrasound with fine needle aspiration (EUS-FNA) of the mass and endoscopic retrograde cholangiopancreatography (ERCP) with the placement of a 10 F x 7 cm straight plastic stent. Endoscopic ultrasound revealed several periaortic lymph nodes, which were not amenable to sampling, the largest measuring 16 x 15 mm, multiple small peripancreatic hypoechoic and round lymph nodes measuring 6 mm in diameter, and a dilated common bile duct measuring up to 13.5 mm in diameter, which was proximally obstructed by a 68.5 x 42 mm hypoechoic, lobulated mass, which was then sampled via FNA. Due to persistent cholestasis, the plastic stent was replaced with a fully-covered 10 x 80 mm metallic self-expandable biliary stent. Following the stent exchange, the patient continued to have persistent cholestasis, and MRCP was performed to evaluate the location of the stent, which revealed the stent to be in the correction position and significant improvement in biliary dilation. Total bilirubin levels began to downtrend and the patient showed symptomatic improvement. Laboratory parameters on admission and at the time of discharge are shown in Table 1.

**Table 1:** Laboratory parameters on admission and at the time of discharge This table shows improvement in liver chemistries, specifically total and direct bilirubin and alkaline phosphatase, after endoscopic retrograde cholangiopancreatography (ERCP) with metallic self-expandable biliary stent placement ALP: alkaline phosphatase; ALT: alanine aminotransferase; AST: aspartate aminotransferase; BUN: blood urea nitrogen; WBC: white blood cells

Laboratory values (units)	On admission	On discharge
WBC (th/uL)	11.9	18
Hemoglobin (g/dL)	9.1	8.3
Platelet count (th/uL)	170	193
Sodium (mmol/L)	135	135
Potassium (mmol/L)	5.3	3.8
Chloride (mmol/L)	105	100
BUN (mg/dL)	89	52
Creatinine (mg/dL)	6.6	4.2
Calcium (mg/dL)	8.9	7.6
Glucose (mg/dL)	152	116
AST (IU/L)	124	131
ALT (IU/L)	105	170
ALP (IU/L)	525	337
Total bilirubin (mg/dL)	32.8	13.8
Direct bilirubin (mg/dL)	17.8	7.7

**Figure 2:**
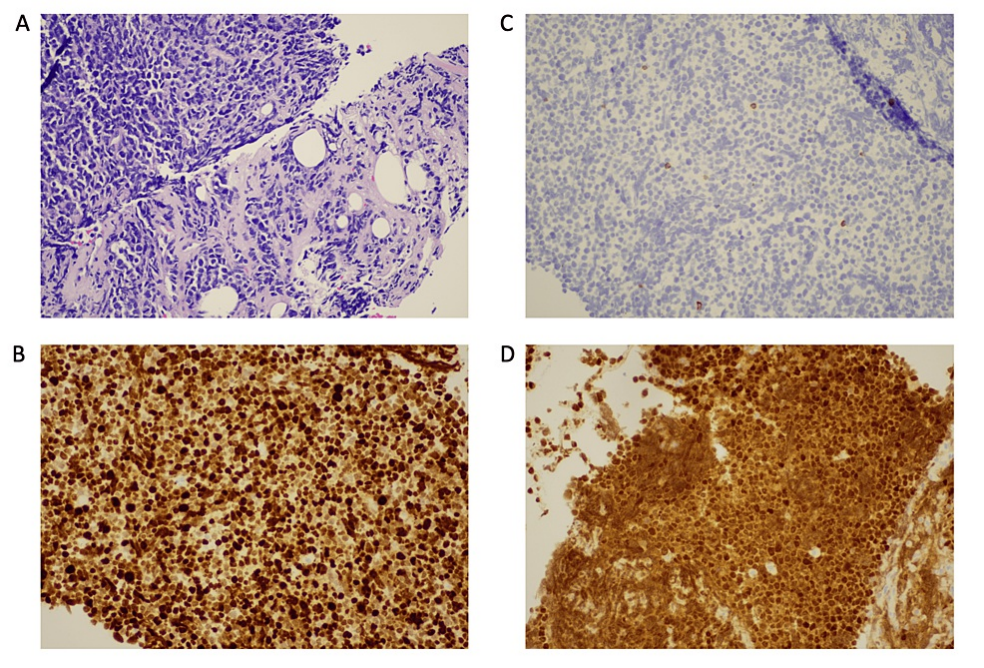
Immunohistochemical analysis of pancreatic mass biopsy Representative photomicrographs of the pancreatic mass showing (A) hematoxylin and eosin (H&E) stain, (B) CD5, (C) CD20, and (D) MUM1 at 20X magnification

Microscopic evaluation of the pancreatic mass showed diffuse sheet-like aggregates of lymphoid cells with coarse nuclear chromatin, moderate pleomorphism, and brisk mitotic activity, which were positive for CD3, CD5, MUM1, and BCL2 and negative for B-cell markers CD20 and PAX5. The cells were also negative for EMA, TdT, CD10, CD30, CD34, chromogranin A, synaptophysin, ALK, BCL6, and cyclin D1. Furthermore, these cells showed a very high Ki-67 proliferation index of >99%. In summary, these findings were most consistent with mature T-cell lymphoma not otherwise specified (NOS) (Figure 2). The patient was discharged home with the plan to start chemotherapy with rituximab, doxorubicin, cyclophosphamide, vincristine, and prednisolone (R-CHOP). The result of the treatment was unknown as there was no record of the patient's follow-up appointment.

## Discussion

Extra-nodal non-Hodgkin's lymphomas (NHLs) account for almost 40% of all cases of NHLs. These tumors are usually located in the GI tract [1]. PPL is a difficult-to-recognize disease, in which presentation and radiologic imaging (e.g., pancreatic head involvement) may overlap with other neoplastic pancreatic diseases [3]. Its common clinical manifestations include abdominal pain, jaundice, acute pancreatitis, small bowel obstruction, and diarrhea. Interestingly, patients do not usually manifest B symptoms (i.e., fever, night sweats), like in the patient presented in this case.

Recently, a study involving a cohort of 39 PPL patients revealed laboratory findings that may suggest PPL. For example, elevated LDH levels associated with normal CA 19-9 levels may indicate a diagnosis of PPL [4]. In another study, CEA levels, often found to be elevated in gastrointestinal cancers such as colorectal cancer and intrahepatic cholangiocarcinoma, were found to be elevated in up to 20% of patients [5]. It is important to remember that the patient described herein had low levels of CA 19-9 and elevated levels of CEA. The most common subtype of PPL is diffuse large B-cell lymphoma (DLBCL), and chemotherapy with R-CHOP-based regimens has proven to be effective in almost all subtypes [6]. Imaging studies including CT abdomen, EUS, and transabdominal ultrasound are all important to evaluate the lesion. There are certain radiological findings that differentiate PPL from pancreatic adenocarcinoma. Pancreatic ductal dilation does not usually occur with PPL, even with proximal ductal invasion, whereas with pancreatic adenocarcinoma, there is distal ductal dilation when proximal invasion has occurred. Lymph node involvement below the level of the renal veins was another finding not associated with adenocarcinoma [7].

Although the above-mentioned imaging findings may indicate a PPL, it is impossible to provide a definitive diagnosis without a histopathologic analysis. While invasive procedures such as laparotomy/laparoscopic techniques may be employed, less invasive techniques, such as a core needle biopsy under EUS guidance, are the more feasible approaches for yielding a tissue diagnosis [8]. A sample may be taken either from the pancreatic mass or the surrounding lymph nodes. Since there are several histological subtypes of this disease, an accurate diagnosis is required. The most common subtypes of PPL include DLBCL (53.6%), follicular lymphoma (9.8%), Burkitt lymphoma (7.5%), and T-cell NHL (6.7%) [3]. 

Chemotherapy and radiation are the primary modalities of treatment, and R-CHOP-based regimens are the most effective; surgical resection alone has not shown survival benefits in these patients. It has been reported that combined chemotherapy with radiation leads to increased survival compared with each treatment modality alone [9]. The prognosis for PPL is higher than the 12.5% five-year survival rate for pancreatic adenocarcinoma [10]. However, recurrence rates are noted to be as high as 24-53% [3,5]. Some authors describe a good response when a hematopoietic stem cell transplant is done following the completion of chemotherapy [11]. It is important to promptly recognize this type of malignancy, which is potentially treatable by chemotherapy and has a better prognosis compared to pancreatic adenocarcinoma.

## Conclusions

It is important to differentiate PPL from pancreatic adenocarcinoma since its treatment and prognosis differ considerably. Some findings such as a large pancreatic mass that does not cause dilation of the main pancreatic duct, absence of hepatic/splenic involvement, and normal CA 19-9 favor a diagnosis of PPL. The use of EUS-FNA is critical in order to establish tissue diagnosis. The treatment consists of R-CHOP-based regimens, which are associated with survival rates of up to 30%.
